# Supplementary material for: Solving for ambiguities in radar geophysical exploration of planetary bodies by mimicking bats echolocation
Source: Nat Commun. 2017 Dec 21;8:2248. doi: 10.1038/s41467-017-02334-1 (PMC5740182; doi:10.1038/s41467-017-02334-1)
Supplement: Supplementary file 1 — Supplementary Information [file 41467_2017_2334_MOESM1_ESM.pdf]

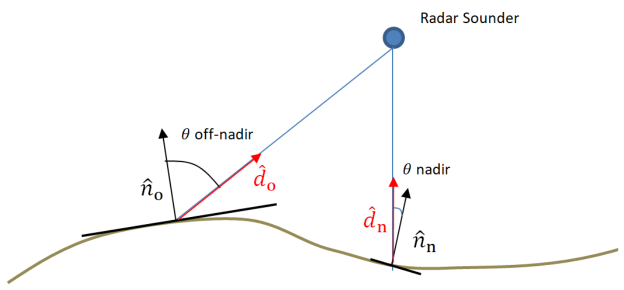

Supplementary Figure 1. Geometry Scheme for the Local Surface Slope Analysis The figure versor definitions is the following: $\hat{d}_n$ and $\hat{d}_o$ are the distance versors pointing from any generic nadir and off-nadir surface location to the radar respectively. Similarly, $\hat{n}_n$ and $\hat{n}_o$ are the local surface normal (i.e. the versor perpendicular to the local surface orientation) to any given nadir and off-nadir surface point respectively.

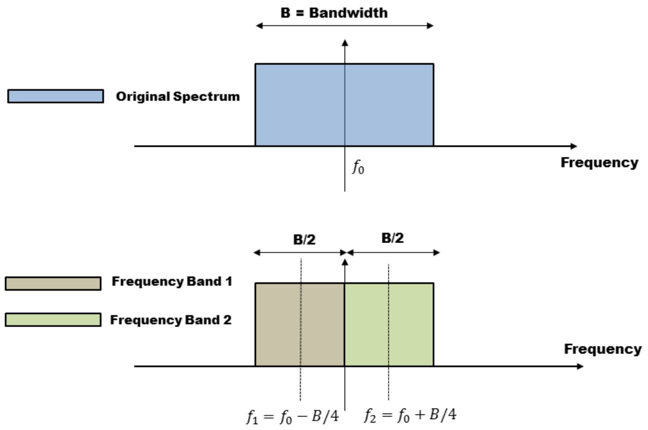

Supplementary Figure 2. Sub-bands processing. Working Principle of dividing the radar bandwidth into two sub-bandwidths

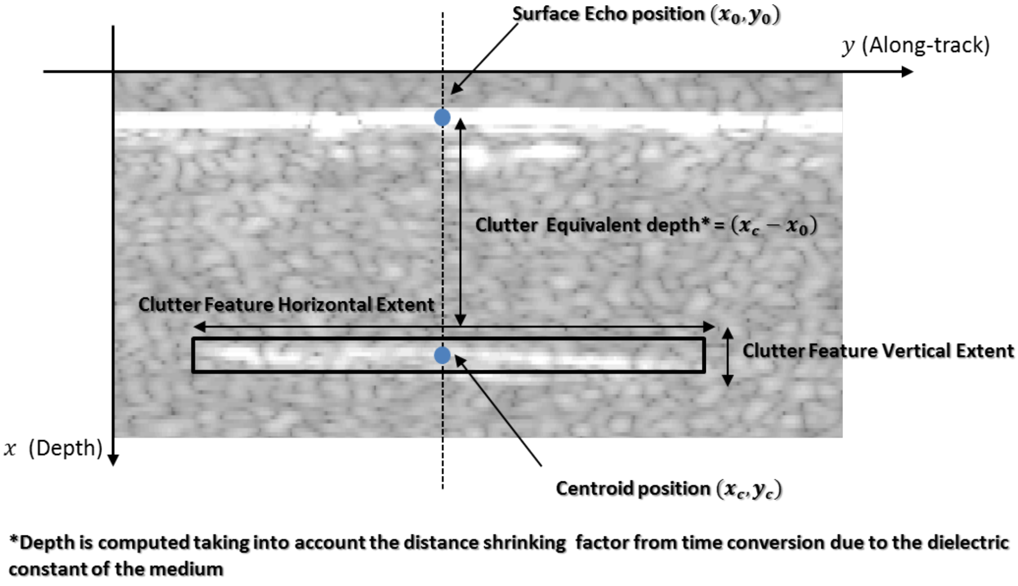

Supplementary Figure 3. Feature Definition and Depth Retrieval. Both clutter and legitimate subsurface features have an horizontal and vertical spatial extent. This allows to compute its centroid and then estimate its equivalent depth in the medium by knowing the surface echo position.

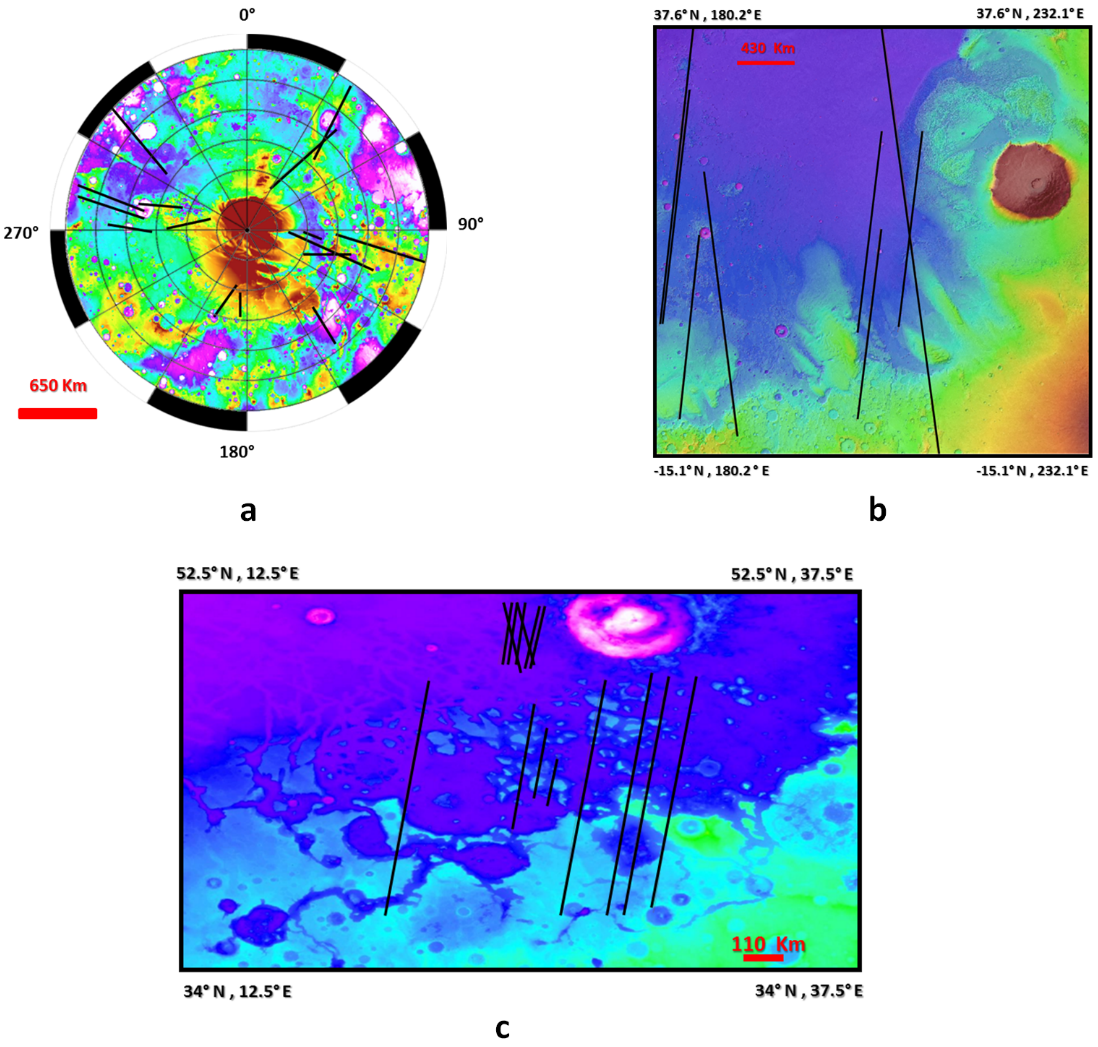

Supplementary Figure 4. SHARAD Orbit Traces over Mars. SHARAD orbit traces depicted as black lines over different Mars regions. These traces were selected for evaluating clutter detection performance for **(a)** South Polar **(b)** Amazonis Quadrangle and **(c)** Deuteronilus Mensae.

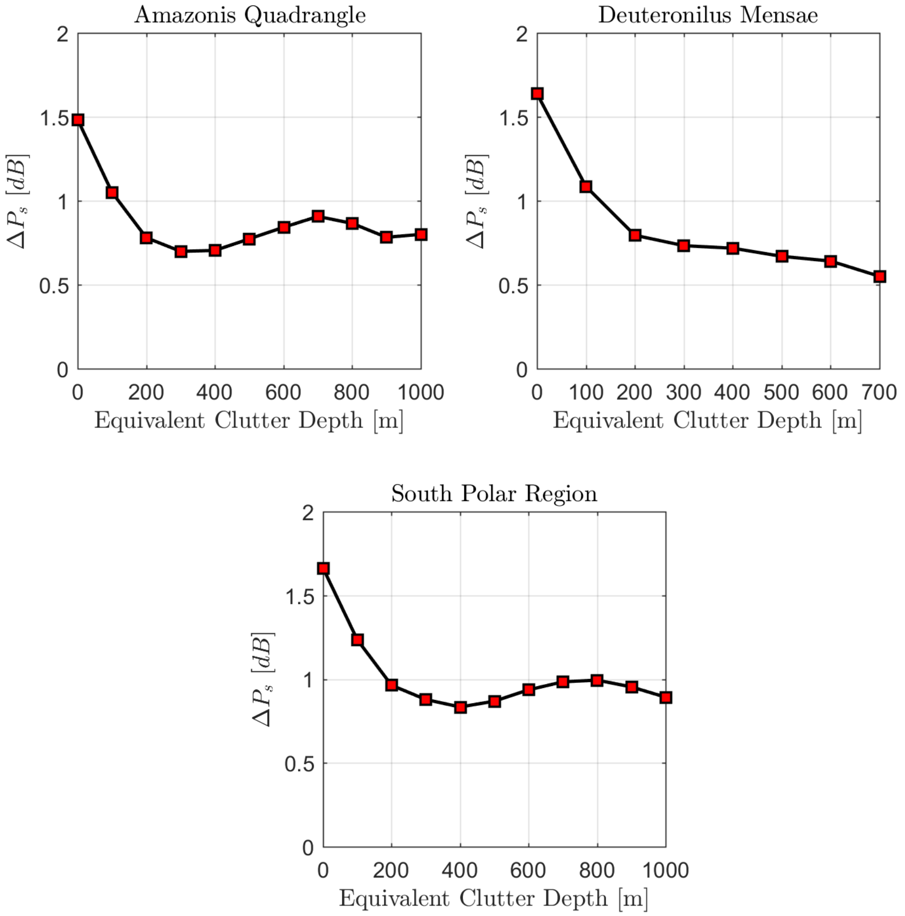

Supplementary Figure 5. Experimental Values of Surface Power Ratio ΔP_s as a Function of Equivalent Clutter Depth. Mean values of the surface power ratio ΔP_s for the considered datasets. Statistics have been computed from radargrams listed in Supplementary Tables 2, 3 and 4 and marked as C (clutter) in the feature type. It is interesting to note that we obtain very similar curves for different datasets.

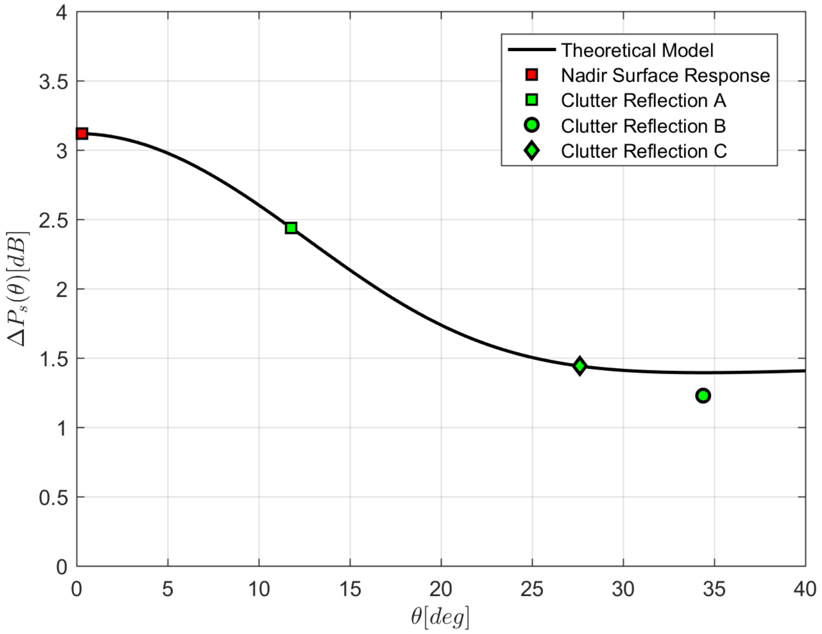

Supplementary Figure 6. Comparison of $\Delta P_s(\theta)$ Values Obtained from the Theoretical Model and from the Experimental Data for the Example of Radargram 0263001 Model theoretical value versus the angle θ for $\Delta P_s(\theta)$ (black line) and comparison with the experimental data (average value). The model parameters for $\Delta P_s(\theta)$ are $f_1 = 17.5$ MHz, $f_2 = 22.5$MHz and $H_s = 0.7$. The results show that the clutter returns of radargram 0263001 (Fig. 7), generated from different craters, correspond to large local slopes relative to the radar sounder. The clutter return of point C, compared to the one of point B, has a smaller local slope with respect to the radar even if it appears at a larger subsurface depth in the radargram. To disambiguate clutter it is sufficient that the nadir surface power ratio is greater than any other clutter reflection coming from off-nadir.

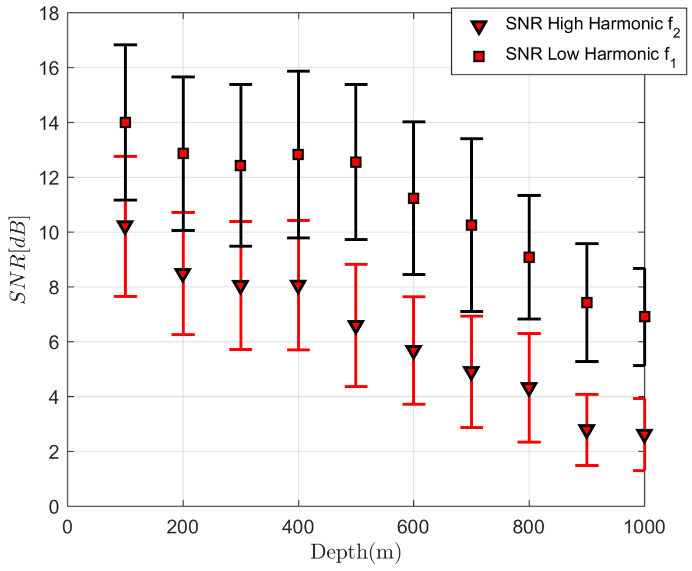

Supplementary Figure 7. Signal to Noise Ratio (SNR) for High and Low Harmonics for the SP Region Subsurface Power Ratio Mean and Standard deviation experimental values of the SNR versus depth for high and low harmonics in the SP. The statistics were computed using the radargrams marked as SS Feature Type in Supplementary Table 2.

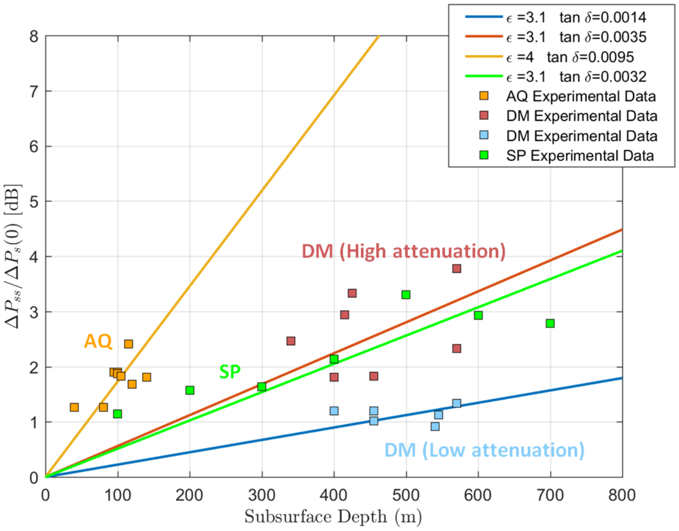

Supplementary Figure 8. Model Prediction of $\Delta P_{ss}(z)/\Delta P_s(0)$ and Comparison with the Obtained Experimental Values for South Polar Region (SR), Deuteronilus Mensae (DM) and Amazonis Quadrangle (AQ) dataset. $\Delta P_{ss}/\Delta P_s(0)$ model predictions (see equation (13)) and comparison with the experimental results for the different datasets assuming $c_h \sim 0$. The disambiguation condition on subsurface signal predicts that $\Delta P_{ss}(z)/\Delta P_s(0) > 0$. For the AQ dataset we obtain subsurface power ratio values that are in agreement with the proposed model assuming a tangent loss of $9.5 \cdot 10^{-3}$ and $\varepsilon = 4$ for the subsurface material. The DM dataset data is divided into two different classes, namely high attenuation and low attenuation. The experimental data are in agreement with the proposed model assuming an icy subsurface with $\tan\delta = 3.5 \cdot 10^{-3}$ for the high attenuation scenario and $\tan\delta = 1.4 \cdot 10^{-3}$ for the low attenuation scenario and $\varepsilon = 3.1$ for both cases. For the SP region, the model predicts the experimental data assuming $\tan\delta = 3.2 \cdot 10^{-3}$ and $\varepsilon = 3.1$. The DM and SP results are consistent with the fact that both regions have similar subsurface properties (i.e. icy regions).

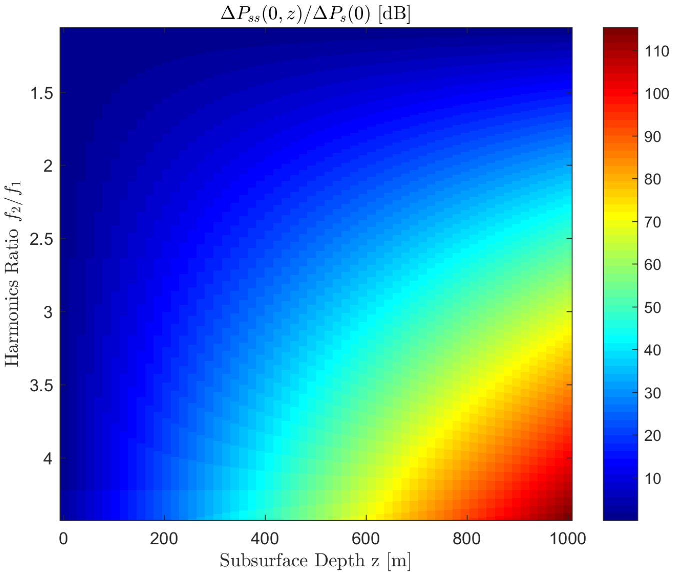

Supplementary Figure 9. Nadir Subsurface Power Ratio vs Nadir Surface Power Ratio. Values of $\Delta P_{\mathrm{ss}}(0,z)/\Delta P_{\mathrm{s}}(0)$ for a representative example of the Mars surface (e.g. Deuteronilus Mensae) assuming tangent loss equal to $\tan\delta = 0.006$ and real part of the dielectric constant of the subsurface medium equal to $\varepsilon_1 = 3.1$. From the figure, it is clear that the method sensitivity improves by increasing the harmonics ratios (i.e. f_2/f_1).

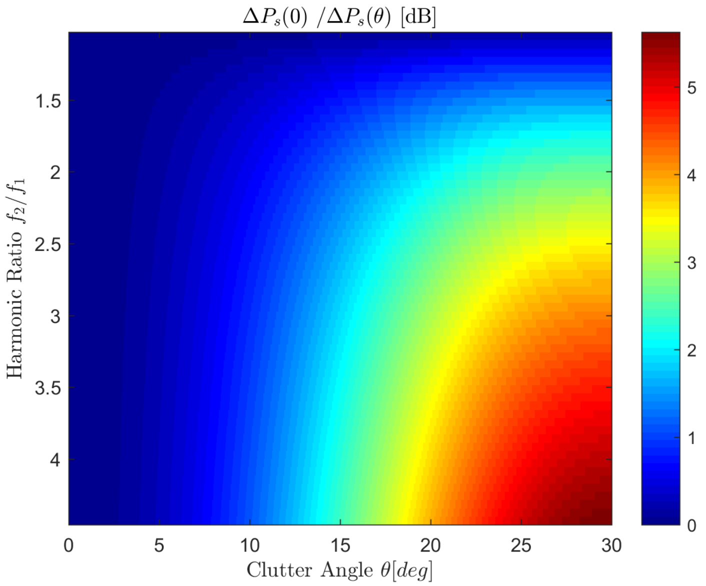

Supplementary Figure 10. Nadir Surface Power Ratio vs Off-nadir Surface Power Ratio. Value of $\Delta P_\mathrm{s}(0)$ /$\Delta P_\mathrm{s}(\theta)$ for a representative example of the Mars surface(e.g. Deuteronilus Mensae). The plot has been computed assuming topothesy equal to $T = 10^{-4}$ m and Hurst exponent equal to $H_\mathrm{s} = 0.85$. From the figure, it is clear that the method sensitivity improves for larger harmonics ratios (i.e. f_2/f_1).

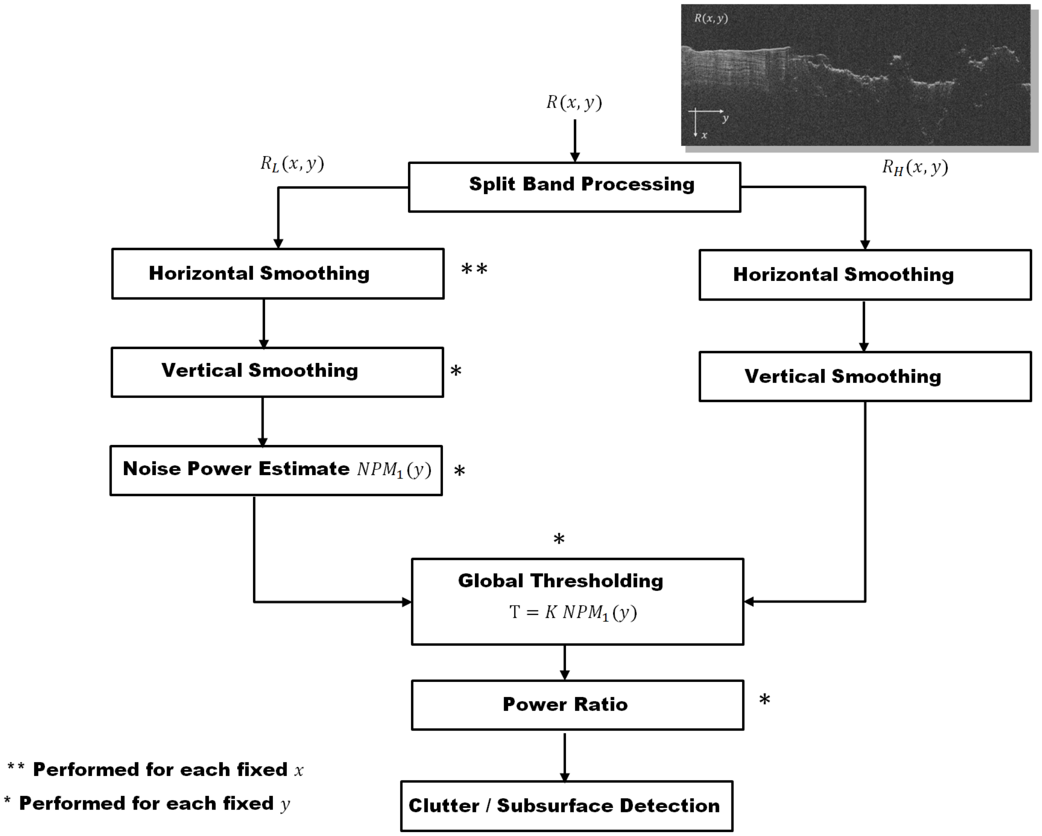

Supplementary Figure 11. **Data Processing.** Processing Pipeline for denoising and evaluation purposes.

Parameter	Value
Nominal Science Orbit Altitude	255-320 Km
Centre Frequency	20 MHz
Chirp Bandwidth	10 MHz
Pulse Width	85 μs
Penetration Depth	Up to 1 Km

Supplementary Table 1. Sharad Main Parameters. SHallow RADar main system parameters

SHARAD RDR data product ID	Feature Type
0176401	C
0216501	C
0219601	C
0220201	C
0246101	C
0263001	C
0267201	C
0284101	C
0459501	C
0614701	C
0659902	C
0747501	C
0754201	C
0764101	C
0776601	C
1204501	C
0241301	SS
0435701	SS
0444401	SS
0459401	SS
0656701	SS
0661202	SS
0770601	SS
0848401	SS

Supplementary Table 2. Data Products IDs for the South Polar Region (SP). SHARAD data products used for testing the proposed model disambiguation condition for the SP dataset. Feature type C indicates a clutter signal only radargram while SS subsurface signal only radargram.

SHARAD RDR data product ID	Feature Type
0308101	C
0470301	C
0478201	C
0484801	C
0527002	C
0556001	C
0562603	C
0598202	C
0628502	C
0647003	C
0685201	C
0722801	C
0740602	C
0770902	C
0772901	C
0856601	C
0683902	SS+C
0690502	SS
0698403	SS+C
0716902	SS+C
0752502	SS+C
0755102	SS
0769602	SS+C
1043201	SS
1232403	SS+C
1260101	SS+C
1305601	SS
2442901	SS+C

Supplementary Table 3. Data Products IDs for the Deuteronilus Mensae Region (DM). SHARAD data products used for testing the proposed model disambiguation condition for the DM dataset. Feature type C indicates clutter signal only radargram, SS subsurface signal only radargram and SS+C mixed case radargram.

SHARAD RDR data product ID	Feature Type
0263901	C
0278501	C
0399802	C
0577101	C
0911301	C
0921303	C
1662901	C
2179901	C
0358901	SS
0523701	SS+C
0526302	SS
0529001	SS
0529001	SS
0530301	SS+C
0556701	SS
0556701	SS+C
0579101	SS
0579101	SS
0721501	SS

Supplementary Table 4. Data Products IDs for the Amazonis Quadrangle (AQ). SHARAD data products used for testing the proposed model disambiguation condition for the AQ dataset. Feature type C indicates clutter signal only radargram, SS subsurface signal only radargram and SS+C mixed case radargram.
